# Decentralized clinical trials and rare diseases: a Drug Information Association Innovative Design Scientific Working Group (DIA-IDSWG) perspective

**DOI:** 10.1186/s13023-023-02693-7

**Published:** 2023-04-11

**Authors:** Mercedeh Ghadessi, Junrui Di, Chenkun Wang, Kiichiro Toyoizumi, Nan Shao, Chaoqun Mei, Charmaine Demanuele, Rui (Sammi) Tang, Gianna McMillan, Robert A. Beckman

**Affiliations:** 1 Research and Early Development Statistics, Bayer U.S. LLC, 100 Bayer Boulevard, Pharmaceuticals, Whippany, NJ 07981 USA; 2 Global Product Development, Pfizer Inc, Cambridge, MA 02139 USA; 3 Biostatistics department, Vertex Pharmaceuticals, Inc, 50 Northern Avenue, Boston, MA 02210 USA; 4 Statistics & Decision Sciences Department, Janssen Pharmaceutical K. K, 5-2, Nishi-kanda 3- chome, Chiyoda-ku, Tokyo, 101-0065 Japan; 5 Biostatistics, Moderna, Inc, 200 Technology Square, Cambridge, MA 02139 USA; 6 Global Biometrics and Data Sciences, Bristol Myers Squibb, Berkeley Heights, NJ 07922 USA; 7 Clinical Development, Global Biometric Department, Servier pharmaceuticals, 200 Pier Four Blvd, Boston, MA 02210 USA; 8 Bioethics Institute at Loyola Marymount University, 1 LMU Drive, Los Angeles, CA 90045 USA; 9 Lombardi Comprehensive Cancer Center and Innovation Center for Biomedical Informatics, Georgetown University Medical Center, Washington, DC 20007 USA

**Keywords:** Decentralized Clinical Trials, Clinical Trial, Pilot Study, Rare Disease, Real World Data, Digital Health Technologies, Advanced Analytics

## Abstract

**Background:**

Traditional clinical trials require tests and procedures that are administered in centralized clinical research sites, which are beyond the standard of care that patients receive for their rare and chronic diseases. The limited number of rare disease patients scattered around the world makes it particularly challenging to recruit participants and conduct these traditional clinical trials.

**Main body:**

Participating in clinical research can be burdensome, especially for children, the elderly, physically and cognitively impaired individuals who require transportation and caregiver assistance, or patients who live in remote locations or cannot afford transportation. In recent years, there is an increasing need to consider Decentralized Clinical Trials (DCT) as a participant-centric approach that uses new technologies and innovative procedures for interaction with participants in the comfort of their home.

**Conclusion:**

This paper discusses the planning and conduct of DCTs, which can increase the quality of trials with a specific focus on rare diseases.

**Supplementary Information:**

The online version contains supplementary material available at 10.1186/s13023-023-02693-7.

## Background

Millions of patients around the world suffer from more than 7,000 different rare diseases that have few or no regulatory-approved standards of care [1]. Limited knowledge about disease origin and variability, and uncertainty regarding clinically relevant endpoints, inhibit design of optimal clinical trials and programs. Small numbers of rare-disease patients who are scattered around the world do not easily produce high-quality, high-density data, which imposes immense hurdles for pharmaceutical companies who must recruit and retain trial participants to generate meaningful results. The expected hardship from traveling to study sites, frequency of specimen collection and health measurements, and communication logistics can dissuade many of these potential participants from joining any kind of research. Loss of this valuable rare-disease participant is a major obstacle for advancement of knowledge that leads to cures.

Participants in traditional clinical trials must visit specific trial sites, which complicates their normal life activities. These additions to their daily schedule can be particularly difficult for children, the elderly, physically and cognitively impaired individuals who require transportation or caregiver assistance, or participants who live in rural or remote locations. Financial costs (e.g., travel, missing work, dependent care), commute time, and site-visits that conflict with work and family obligations, often preclude participation or reduce compliance. Tests and procedures for research, over and above treatment already received for their chronic disease, may be prohibitively burdensome. There is evidence that virtual participation in clinical trials from patients’ natural habitat, using their native language via digital applications, can facilitate recruitment, compliance, and retention of participants [2].

A Decentralized Clinical Trial (DCT) is a patient-centric approach that uses new technologies, advanced analytics platforms, and/or innovative procedures to communicate with patients and participants in their homes or native environment. These technologies include medical telecommunication, digital health technologies (DHTs), deliveries of clinical supplies, shipment of collection of specimens, and remote supervision [3, 4]. While some tests, such as magnetic resonance imaging (MRI), as well as high quality physical exams, require physical presence at a site, a hybrid approach can still significantly reduce hardship on trial participants and their families. Moreover, DCTs can increase enrollment and population diversity that is currently lacking in rare disease trials, and thus increase the generalizability of trial results [5, 6]. The Clinical Trials Transformation Initiative defines DCTs as those trials that are “executed through telemedicine and mobile/local healthcare providers using procedures that vary from the traditional clinical trial model” [7]. These studies are augmented by the use of DHTs that collect physiological data such as physical activity and vital signs [8].

DCTs can reduce the frequency of visits, increase the flexibility of visit schedules, decrease the cost of the trial, and potentially enhance the quality of behavioral monitoring data and participant compliance. Participation in research from a home environment can prevent unnecessary exposure to potential and contagious diseases, especially for children who cannot be vaccinated due to fragile health status or during epidemic or pandemic. In fact, during the COVID-19 pandemic, an American College of Rheumatology guidance for the management of children with pediatric rheumatic disease during the COVID-19 pandemic states that “shared decision-making should occur between patients, families, and rheumatology providers to discuss additional measures to reduce interruptions in clinical care, particularly during periods of increased community transmission” [9]. Such measures include use of telemedicine for routine, regularly scheduled, and nonurgent clinical assessments, and physical therapy [10].

Pioneers who have successfully conducted DCTs include Pfizer, Inc, who in 2011, sponsored the “Web-based Methodology Trial to Evaluate the Efficacy and Safety of Tolterodine ER in Subjects With Overactive Bladder (REMOTE)” trial, an exploratory, randomized, double-blind, placebo-controlled, parallel-group, single-center, Phase 4 trial, to test a novel web-based trial design for evaluating the efficacy and safety of tolterodine extended release 4 mg in US participants with overactive bladder [11] (NCT01302938). As described in the report, “Participants were recruited via the web, screened for eligibility using web-based questionnaires, had laboratory testing in their community, and entered a run-in phase requiring bladder e-diaries. Informed consent was obtained using an interactive web-based method with physician countersignature. Study medication was shipped directly to participants.” [11]. As the first entirely web-based trial conducted under an Investigational New Drug (IND) application, the efficiency and results shown in the study were consistent with results from traditional centralized clinicals trials.

Pfizer Inc. also recently launched the “Study Evaluating Efficacy and Safety of Crisaborole in Adults with Stasis Dermatitis, a Phase 2, Randomized, Double-Blind, Vehicle-Controlled, Proof-of-Concept Study to Evaluate the Efficacy, Safety, and Local Tolerability of Crisaborole Ointment, 2%, in Adult Participants with Stasis Dermatitis Without Active Skin Ulceration” as a decentralized study (NCT04091087). Enrollment and management were decentralized. The sponsor (or designee) provided home visits by qualified mobile healthcare professionals (HCPs), remote contact by telemedicine or virtual visits, and clinical database electronic case report forms (eCRFs), eDiary, and other electronic data entries from third party vendors for study data collection.

The COVID-19 pandemic inspired clinical researchers to consider switching to DCTs. In 2020, during the height of COVID-19, University of Minnesota sponsored the study “Post-exposure Prophylaxis / Preemptive Therapy for SARS-Coronavirus-2 (COVID-19 PEP)”, a multisite, international, randomized, double blind, placebo-controlled trial with a parallel design to investigate whether hydroxychloroquine could reduce COVID- 19 severity in adult outpatients [12] (NCT04308668). In response to quarantine measures and evolving social-distancing rules, all aspects of this trial (such as screening, drug shipment, data collection) were conducted virtually. Instead of scaling back or pausing ongoing studies, Vertex Pharmaceuticals embraces COVID-19 restrictions to pilot a fully decentralized clinical trial (NCT04923464). This Phase 4 study was performed to learn more about the data that wearable technology can provide about physical activity, cough frequency and sleep quality in people with cystic fibrosis while taking commercial Elexacaftor/Tezacaftor/Ivacaftor. All visits were home based and were conducted via telemedicine video conference via a mobile app [13, 14]. A list of other DCTs with information released on clinicaltrial.gov can be found in Table A1 in Appendix A. These concrete examples are intended to inform the development of future DCTs.

In this paper, we discuss the major components of and considerations for DCTs. A list of examples of DCTs is offered, highlighting key data capture points. We hypothesize that DCTs are an innovative and patient centric way to reduce the burden of clinical trials on patients, caregivers, and clinical researchers, especially for the rare disease population. We depict a road map to DCT while focusing on components of DCTs that require specific considerations and customization for pediatric patients and/or patients with rare diseases. The suggestions are based on a combination of those examples of DCTs and the DIA-IDSWG perspective. Our team includes statisticians, a bioethicist, a physician/clinical researcher, and several patients and caregivers.

## DCT models and components

Traditional clinical trials require all participant visits to be on-site, while fully decentralized clinical trials require all visits to be conducted remotely via telemedicine. For example, trials that require surgery, cell or gene therapy, or MRI imaging, require participant presence at the trial site. Studies involving data collection such as monitoring basic vitals or collecting skin images, allow for remote participation. A DCT does not always require a fully virtual approach. A hybrid model combines traditional on-site visits and remote visits. Sponsors can recruit mobile HCPs for measurement or intervention tasks requiring special training. Within a single trial, some participants may be enrolled at traditional clinical trial sites, while others may be enrolled or managed in a decentralized or remote manner, according to their needs and preferences [7]. A hybrid approach reduces participant burden and can be implemented across all phases of a study. Table 1 summarizes different components of a clinical trial that could be virtual and/or in person. These choices are made by the study team and a pilot study is recommended [15].

## A road map to DCT

**Fig. 1:**
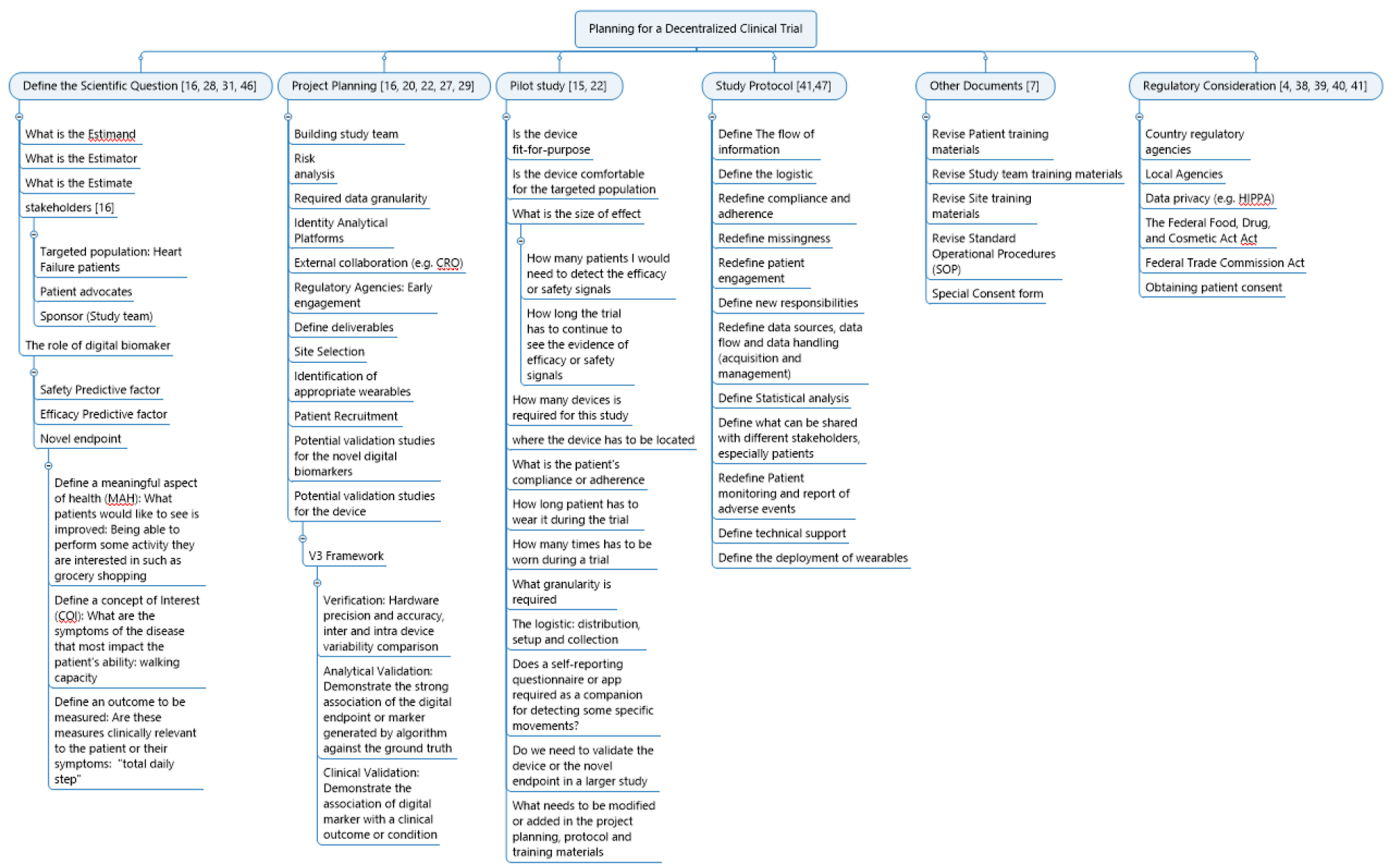
Flowchart - Key Steps for Conducting DCT

A comprehensive summary for key steps to consider can be found in Fig. 1, which can be used as a checklist for clinical trial researchers and sponsors to plan, conduct, analyze, and report a DCT. In this section, suggestions are offered for the success of a DCT with a focus on elements and landscapes that are relevant to rare disease and different from a traditional, centralized clinical trial which calls for specific attention and customization.

### Stakeholders and project planning

A DCT design must identify and involve all stakeholders and develop study-specific communication logistics [16]. Examples of internal stakeholders include but are not limited to the clinical research team (clinicians, statisticians, clinical data scientists, project leads, etc.), regulatory affairs and legal team, and patient out-reach team (such as communication, marketing, training). Close collaboration is required among cross-functional teams to modify traditional study practices to DCT formats. An initial evaluation of internal skills and experiences will indicate what external collaboration and outsourcing are required to move forward efficiently. Depending on the study timeline and size of the company, some tasks might require outsourcing to an experienced third party.

External stakeholders including sponsors, patients and caregivers, patient advocates, clinicians, regulatory authorities, private payers, contract research organizations (CROs), DHT vendors, academic researchers, mobile or local HCPs, professional medical associations, and technology companies. The concerns and priorities of these stakeholders must be addressed during the design of the DCT study. The supervisory responsibilities of the investigators and the authority for remote site monitoring must be established. Cross-industry coalitions such as the Decentralized Trials and Research Alliance can help support reducing risk and promoting and improving the conduct of a DCT [17]. The Clinical Trials Transformation Initiative is another resource that can identify legal, regulatory, and practical barriers to conducting DCTs and identify opportunities to clarify and inform policies that affect the implementation of DCTs.

Planning begins with questions related to a broad context of a proposed DCT (Table B1 in Appendix B). Next, the core team defines the components of a DCT, administrative and research roles and responsibilities, internal and external required skills, technologies, and regulatory compliance. In rare diseases it is crucial to capture patient and caregiver perspectives during the trial design [18, 19].

A pre-trial feasibility study can evaluate the operational barriers, selected strategies, staff comfort level, and patients’ acceptance of this new approach. An example of successful modification of a centralized study to DCT is REMOTE [11] mentioned in Sect. 2, the first web-based study conducted under an IND application. It was designed to replicate previous clinic‐based trials of tolterodine extended release. The REMOTE study is remarkable in that it illustrates both the challenges and benefits of digitizing a clinical trial. Such pre-trial feasibility studies may be challenging to conduct for rare diseases due the rare number of patients, however, a feasibility study in a similar population can provide meaningful insight.

### Site selection and clinical operation

CROs and sponsors may be asked to fulfill novel roles as technology partners for patients and must be willing to adapt to the new approach [20]. Sites that embrace DCTs need new operational frameworks and additional support from Sponsors and CROs as they navigate this new research environment. It is important to review and understand site-specific telemedicine laws and mandated systems for tracking information, such as receipt and drug accountability in remote trials.

It is difficult to recruit patients with a specific rare disease in numbers large enough to produce meaningful results in a clinical trial. If these patients are scattered over a wide geographical area (even internationally), it is extraordinarily difficult to bring them to physical trial sites for specialized study interventions. The flexibility of a DCT allows for remote tasks and data collection, which reduces the responsibilities and limitations of a potential local sites, allowing for a larger trial network which can serve a greater number or participants.

The majority of DCT site operations are similar to that of traditional clinical research, but require modification that addresses new technology, internet availability, and interaction with participants. Clinical staff must be trained in these new processes. If a novel DCT is planned and/or the indication or target population is different from a previous DCT, early engagement can be simple with advanced interaction introduced gradually. A DCT can be a reasonable choice for early phase trials since internal decisions involve smaller sample sizes. This allows the study team to evaluate the study design and operational plan as they prepare for later stage pivotal trials and before submission of the DCT protocol to authorities.

### Patient monitoring and safety

Both traditional centralized trials and DCTs must report study related safety events. Sometimes, participants must understand how to access DHT information and communicate this data and any significant health developments to the clinical staff. If the participant is cognitively impaired or has difficulty with technology, it is important to ascertain whether the caregiver will be available to manage the DHTs, connection to remote visits, etc. Participants who do not have reliable internet service may not be able to participate remotely unless the sponsor explicitly provides this. Options must be individualized, and the study team must listen carefully to the preferences and concerns of participants and caregivers. One of the advantages of using DHTs in a DCT, is that continuous monitoring of participants can flag safety issues and adverse events in real-time, allowing investigators to respond in a timely manner. This might be preferable, for example, in pediatric studies or with cognitively impaired patients who cannot communicate or recall the occurrence and details of adverse events on their own. Although early detection of peripheral problems by DHTs is not the primary goal of a DCT, there should be plans for real-time response to such events. In addition, the risk of erroneous measurements should be addressed. Finally, detection of some risk states requires advanced physical examination skills that specialized physical examination skills that are not generally shared by home health personnel. For example, cardiac auscultation by an experienced cardiologist may detect cardiac issues that are not detectable by remote technologies. Conversely, continuous cardiac and/or activity monitoring may be superior to an examination by an experienced cardiologist for detecting other cardiac issues.

As mentioned, a DCT can be fully remote or hybrid with both remote and on-site components. It can be beneficial to have the patients come to an on-site visit in DCT, especially for the baseline visit and/or the study end visit. Clinically vulnerable pediatric patients may require an initial on-site visit for a comprehensive evaluation. There is no “one size fits all” and DCT designs, and individual patient schedules should remain flexible.

### Digital health technologies

In 2017 and 2018, over 1100 unique trials included the use of DHTs as compared to only eight trials in 2000 [21]. COVID-19 accelerated this shift. Stakeholders now realize the advantages of this approach, especially in the case of extremely ill patients, children, and rare disease populations. Continuous data collection using DHT in free-living environments allow the capture of a new category of objective measurement that illuminates the nature of disease progression and derives clinically meaningful endpoints that were previously impossible.

Data provided by vendors and device companies do not always translate to a specific patient population. When incorporating DHTs into a DCT, it is important to select fit-for-purpose DHTs to quantify a measurable estimator in the targeted population that is based on the study objectives and ensure comprehensive and validated evidence when using digital endpoints. A validation plan integrated into the early stages of clinical development might be necessary. Low numbers of potential participants in rare disease trials might best be saved for a pivotal study rather than a pilot study to test DHTs, but strategies can be borrowed from “drug repurposing” studies, to reduce the time, cost, and risk to deliver a drug candidate for a new indication based on previous work that may include validation of previous DHTs [22]. Similarly, devices and the accompanying novel digital endpoints can be derived and/or validated on a common disease population with similar symptoms/indications to a rare disease. For example, common chronic heart failure and heart failure due to a rare genetic mutation (e.g. LMNA related dilated cardiomyopathy [23]) both share symptoms including fatigue, exercise intolerance, and limitation of activities of daily life [24, 25]. Therefore, if physical activity measured by an actigraphy device has shown to be clinically relevant to disease progression for a common chronic heart failure, it is reasonable to generate such digital endpoints to rare cardiomyopathy.

As suggested previously [26], when deploying DHTs into clinical studies, it is crucial to incorporate patients’ voices, provide comprehensive audio-visual instructions related to DHT use, and develop a support system (e.g., 24/7 technical assistance) for patients and caregivers in case of technical difficulties.

There is a growing amount of guidance on DHT use for remote data acquisition. Examples include the ICON plc white paper for an eight-item checklist [8] and the Digital Medicine Society (DiME)’s proposal of the V3 framework (verification, analytical validation, and clinical validation). The V3 framework evaluates DHTs as fit-for-purpose for use in clinical trials. It is emerging as the gold standard, and has been adopted by the European Medicines Agency (EMA) [27]. DiME also hosts a library of digital endpoints used by registrational clinical trials [28]. A recent work was published to discuss the “bring your own device” (BYOD) option as a more user-friendly strategy to allow patients to use familiar technologies, which can ensure better compliance and unbiased measurements [29]. This is closely related to and applicable for a DCT.

### Data handling and statistical analysis

Infrastructure and advanced analytics platforms are the foundation of technologies for capturing high-density data. Small pharma companies and CROs who focused on orphan drugs or rare diseases can be challenged by the data volume of these technologies. For example, raw accelerometry data, which passively collects patient movement and activities, at the sub-second level can result in millions or even billions of observations during the monitoring periods of weeks and months. Multi-modal data such as audio, image and DHT data may be collected simultaneously, continuously, and longitudinally. Such high volume and complex data require advanced analytic platforms and complex analytical methods that most pharma and CROs running classical trials do not have access to. To address this, pharma companies must allocate funds to set up advanced, flexible environments for analyzing the acquired and cleaned data in house. CROs have already begun establishing platforms for accommodating digital data and gaining experience in handling new technology and data through mergers, acquisition, and collaborations with companies from tech sectors [30, 31].

Such complex data collection may not always be feasible for DCTs targeting a common disease with high prevalence as it will increase the burden of collection and handling of large volume of the data given the large number of participants. However, a DCT targeting a rare disease may benefit from such multi-modal data, since the smaller patient population means a lower data burden and an opportunity for a holistic picture of rare disease patients. This high-volume data may be important for choosing or developing an optimal pivotal endpoint for a rare disease that may be selected adaptively during a pivotal study, averting the need for a separate natural history study to choose a pivotal endpoint [32, 33].

Analytical platforms may store data in different measurement modalities, synchronize data using multiple sensors, and require (nearly) real-time data processing with remote monitoring. This synchronization of data capture is challenging [34]. Sponsors and vendors need to coordinate these functions. For example, AiCure uses mobile apps to monitor whether patients have taken the drug and collect daily adherence data; some actigraph devices can detect compliance issue (device not worn) in real time by enabling data transfer via devices’ Bluetooth to actigraph’s cloud system. Such functionality can ensure the smooth conduct of a DCT.

Similar to a traditional clinical trial, a fit for purpose statistical analysis plan (SAP) needs to prespecify analyses required to achieve study objectives. One particularly important thing to consider is the occurrence of intercurrent events (ICEs), which are events that occur after the initiation of the study intervention that precludes the observed outcome or affects the measurement and can be more complex due to the remote data collection. These call for carefully evaluation and response [31]. For example, in DCTs with remote data collections via continuous DHTs, missing data can be more complicated given the noisy and complex data structure, and the lack of supervision in the free-living environment. The characteristic of missing data from DHTs, emerging statistical and data processing techniques to analyze and impute continuous epoch level and daily summary data are discussed in the literature, and suggestions are provided for preventing missing data from device deployment and trial design [26]. Moreover, novel statistical methods such as functional data analysis, data integration, and robust feature engineering are applicable to derive meaningful summaries from the continuously collected DHT data [35–37].

### Regulatory considerations

When a novel design or methodology is applied to a clinical trials format unfamiliar to regulators, careful negotiation is needed. Because there is no widely used concrete guidance for DCTs, clinical trial sponsors and regulators should work together. The success of a DCT submission hinges on good communication with all stakeholders and fulfillment of regulatory requirements. For example, the FDA published draft guidance of Digital Health Technologies for Remote Data Acquisition in Clinical Investigations in January 2022 [4]. This guidance encourages sponsors to ensure DHT is fit-for-purpose and outlines the information to be included, such as DHT selection, description, verification/validation/usability, clinical novel endpoints, statistical analysis, risk considerations for wearable device, and record protection/retention. Innovative Science and Technology Approaches for New Drugs (ISTAND) Pilot Program can be another source of information and recommends sponsors to consider cybersecurity information available on the FDA’s website. Finally, the EMA guidance on DCTs is ongoing [38].

One particular concern arises in the flow of data collected from a DCT. DCT patients who experience medical issues that require intervention might contact the pharmaceutical company or the sponsor sometimes before the clinicians and/or investigators, due to the commonly used automated data transfer set-up. The pharmaceutical company or the sponsor will then have to share the patients’ requests with the clinicians and/or investigators. This is problematic because regulatory requirements (such as Health Insurance Portability and Accountability Act, a.k.a. HIPPA) require data to be anonymized to sponsors, which means that the clinicians and/or investigators will not be able to know which patient it is. Therefore, it is crucial to emphasize the data flow in the study protocol to make sure patients can contact the institutions directly to avoid raising any ethical or regulatory red flags.

The regulations differ between countries. For example, sponsors must follow national data privacy regulations, such as HIPPA in the US, General Data Protection Regulation in EU and Act on the Protection of Personal Information in Japan. As of December 2022, Japanese regulations to promote DCTs are under discussion. Topics include regulation of the remote consent and data reliability in DCTs, direct shipment of investigational medical product (IMP) to participants, and site personnel resources for visiting participant homes [39]. In Denmark, the investigator delivers IMPs directly to the participants [40]. In each instance, the DCT sponsors should document all logistics in the protocol and be sure of compliance with each regulatory agency [41].

As for DCTs targeting rare diseases, there is yet any detailed regulatory guidance. However, it is critical to think about the challenges (such as the concern about data flow and discrepancy in regulatory requirements on novel technologies between countries/regions, mentioned above) in rare disease clinical trials and investigate novel approaches that have been already offered by regulatory bodies for rare disease domains. The key consideration should be how such novel approaches can be adopted to DCTs. One has a better chance of submission and expedited review if studies are tied to the suggestions of regulatory bodies.

### Ethical considerations

DCTs are more than just convenience for trial participants. Participating in research in a hybrid or completely remote manner allows a participant to spend precious minutes and their limited energy with loved ones or participating in life activities that have no connection to illness. The parent-child bond often suffers from the stress of repeatedly cajoling a child into going to the hospital or allowing strangers to do medical procedures to gather information. It is no little gift to allow parent and child to do as much as possible in the comfort of their own home. This reflects the ethical principle of beneficence, which charges researchers who are in the quest for valid scientific data to “do good” whenever possible.

As stated earlier, there may be trial participants for whom remote participation is not suitable, either due to poor internet service or lack of comfort with technology. Sending a physician to the patient’s home to perform tests that are conventionally done on a clinical site is an immediate quality of life improvement. Patients’ and caregivers’ preferences should be prioritized, and relevant questions include: Have the patient and caregiver demonstrated the ability to participate remotely? What degree of remote participation is consistent with optimal care quality? Hybrid DCTs, with potential individualization, involve active listening by the clinicians involved in study intake, considering the patients’ and caregivers’ preferences as well as the medical issues, and no compromise regarding quality of care. To fully encompass this extension of beneficence, DCT technology must be highly reliable and remote support (as well as on-site support) must be available in the event of technological failures.

DCTs allow people to participate in trials who would not have been able to manage a rigorous onsite schedule. And because of the nature of any given rare disease, eligible participants are geographically scattered. Hybrid or completely decentralized trials allow meaningful research to be done, by pulling together cohorts of participants who are not tied to a centralized physical site. This is in service to the principle of justice – supporting greater access to opportunities to participate in research and buttressing the diversity of the research participant population. As discussed above, certain components of care can be delivered at higher quality at a tertiary center. Most obviously, the trial participant may need to return to the trial center for high technology diagnostic or therapeutic interventions, and these will likely be built into hybrid trial designs when necessary. We have also alluded to a more subtle point: DCTs must incorporate in-person physical examination by experts when remote examination is not possible or feasible. This hybrid model will ensure high quality of care.

Finally, there is a psychological component embedded in learning to monitor your child’s (or your own health) for meaningful data. This personal management of care in the furthering of science – can be empowering. Active participation is the embodiment of autonomy, that is to say– those in a DCT are literally carrying out some of the research procedures themselves. DCT participants are reaffirming their consent to participate with every action they take, and their autonomous actions reinforces the reality of “partnership in research.”

## Conclusion

The COVID-19 pandemic shifted the traditional clinical trials to the more patient-centric decentralized approach, which is necessary and a big step forward. But questions remain. A shortage of home-health care personnel is a major demographic trend that has been exacerbated by the pandemic. The burden that traditional clinical trials impose on patient/participant life was already well understood but addressing those issues requires more than manipulating logistics or finding new ways to “measure things”. It requires a change of mindset, assessment methods, estimand and estimator, and interpretation.

DCTs do not replicate or replace a conventional, centralized, randomized clinical trials (RCT) [42] but reflect the real-world information in clinical trials that was missing before. The researcher’s job is to ensure that the trial processes and results are valid, clear, and informative for all stakeholders, and that quality of care is maintained [43]. With a proper plan, study protocol, and risk-based analysis, and involving regulatory agencies in the process, the majority of the concerns can be addressed [44].

As required by the EU’s Regulation (EC) No. 141/2000 on orphan medicinal products, “patients suffering from rare conditions should be entitled to the same quality of treatment as other patients,” and “it is therefore necessary to stimulate the research, development and bringing to the market of appropriate medications by the pharmaceutical industry” [45]. DCT, as a patient centric approach, provide more possibility for medical treatment research. The validation of novel approaches and pilot studies need to be conducted and emphasized before using DCTs for treatment research.

Although DCTs have potential benefits, we should not overlook the accompanying challenges. For example, careful design and implementation of data collection as well as monitoring and validation of data such as effective digital biomarker [46] is required to ensure the data quality and accuracy. More cautious planning and implementation of data management procedures are needed to ensure data privacy and security as data may be transmitted remotely. Moreover, effective study management and oversight is critical and can be particularly challenging in a decentralized setting to ensure patient engagement and retention. This is particularly crucial in the current stage of DCTs, as clinical trial sponsors, investigators, and regulatory parties are still familiarizing themselves with the components of clinical trials in a decentralized setting [47]. It is also challenging to establish close collaboration between clinical trial sponsors, healthcare providers, and technology vendors to ensure the interoperability and integration of DCTs with existing healthcare systems which have been tied more closely to conventional centralized trials in the past.

DCTs present an opportunity but also a difficult trade-off between the advantages they offer and the need to ensure quality care and rigorous measurement of safety and efficacy. In designing the trial, clinical specialists must consider the severity and complexity of the condition and the aspects of the population, and how the trial can be designed to retain quality care and endpoint assessment. Critical elements of care, including specialized physical exams and procedures, are essential for quality care, and on-site visits should be mandatory when they are medically necessary. Sponsor clinical trialists and health authorities should work together to ensure sufficient rigor of study endpoints. At the same time, members of the patient and caregiver community should have input into study design. These discussions can define which elements of the trial must be done on site, and how DCT elements can be safely incorporated to enhance the trial participant and caregiver experience. It is possible that with time, DCT infrastructure and technologies may improve, allowing more functions to be done remotely without compromising quality. Meanwhile, hybrid DCTs may be a good way to “start small” while this approach is being further developed.

**Table 1:** A list of DCT potential components categorized by the different aspects of a clinical trial

COMPONENTS	FULLY DECENTRALIZED	HYBRID	SPECIAL ATTENTION FOR PEDIATRIC COHORT
**Site activation**			
Site selection and activation	Can be done remotely through teleconference.	Similar to a fully decentralized trial	Appropriate settings for recruiting children may include schools, childcare centers, and other common settings for children. Researchers should ensure that the data collection site is safe, convenient, and child-friendly
**Enrolling trial participants**			
Recruitment	Recruitment can be conducted by web-based methods, social media, physician referrals, patient advocacy groups.	TV, radio, newspaper, physician referrals, posted fliers, mailings, cold calls, and the internet.	Risks and benefits need to be more properly explained (especially when there are no direct benefits)
Consent	Potential participants review required study documents and provide a digital signature for consent remotely. We need to ensure that participants are comfortable interacting with staffs.	Similar to a fully decentralized trial	A dedicated team to address parents’ and children’s question before signing the consent and assent in a timely manner (24 − 7, same day, or at least next day availability)
**Trial conduct**			
Pre-screening and clinical visits	Televisit: Investigator-Participant interactions can be connected by bilateral communication (via the mode of Face-to-face, Telemedicine, Text, Chat, Telephone, etc.) Home health visit: Trained mobile healthcare providers (e.g., nurses, physicians, phlebotomists) conduct in-person evaluations at a time and location convenient to the participant. Mobile healthcare professionals (HCPs) perform safety procedures, such as blood collection, vital sign review and other required study evaluations. Mobile HCPs provide supplies and instruct the participant the ability how to collect urine, saliva, and stool samples in their own home to be sent to the central lab for analysis.	Trials with complex imaging needs, radiographic and surgical procedures (e.g. biopsies) require on-site visits. Remote visits, as appropriate, can be considered for follow-up. Some decentralized activities may only be accepted (e.g. culturally or per local regulations) in some countries and/or patient populations and may necessitate another type of hybrid approach.	For televisit, the investigators need to ensure that the children are willing to compliant with the directions via telemedicine, chat, etc.
Data collection	A clinical outcome assessment (COA) can be administered on a general-purpose computing platform (e.g. mobile phone, tablet, or smart watch) and is then referred to as an electronic COA (eCOA). Types of COA include clinician-reported outcomes, observer-reported outcomes, patient-reported outcomes, and performance outcomes. Continuous monitoring of vital signs and other status assessments by Digital Health Technologies (DHTs) are considered as one of the major data sources. During the home health visit, the mobile HCPs can input participant study data directly into eSource data (e.g., electronic Case Report Form) which feedbacks to the site or directly feed into the Electronic Data Management.	Similar to a fully decentralized trial	eCOA and ePRO need to be fit-for-purpose for the pediatric cohort’s age and knowledge. For example, sometimes emoji-based responses are more interpretable than text-based responses. If DHTs are used, their form factors should be accepted by the pediatric patients. For example, a common size apple watch may be inconveniently big for a preschooler. Sometimes, color watchbands and those with animals’ figures can be more welcome for younger children. A pre-study feasibility and user acceptability test may be called for.
Safety Monitoring	Train investigative staff on processes that are unique to DCTs, such as remote safety monitoring and procedures needed to support documentation. A list of approved local health care facilities and/or clinicians for emergent issues help the participants at a remote location with questions about possible adverse events. Investigators must coordinate facilities/clinicians near the trial participant’s location.	Can be similar to a fully decentralized trial	A 24 − 7 team who understand the common safety concern and safety monitoring should be ready to answer questions. A regular email or text check-in may be called for.
Clinical supply	Samples, supplies, and unused investigational medicinal products (IMPs) can be directly shipped or collected from the participant’s home or to home nursing agencies, which can be managed by investigational products accountability tracking system.	Trials with complex investigational product administration (e.g. gene therapy) require on-site visits	Children’s school and other care-related schedules need to be considered when an on-site visit is called for.
Trial closeout	eTMF (electronic Trial Master File) is the trial master file in a digital format. The EMA and the FDA have released regulations, policies, and guidelines to follow for validating the use of this electronic format, the most widely followed being CFR 21 Part 11.	It has become standard in the pharmaceutical and biotechnology industries to use an eTMF, a good option for both hybrid trials and traditional trials.	eTMF is the standard practice.
Data monitoring	Tools of centralized monitoring (e.g. remote evaluation of clinical data) can be developed to identify key quality and risk indicators early (e.g. missing data status) and monitor them throughout the study. Remote source document review provides site monitors remote access to physical sites so that they can continue to monitor source data and documents. Clinical research associates (CRAs) can work remotely, even completing source document review or full site visits from home using the same types of technology that patients use for virtual visits.	Centralized monitoring is still a critical component for hybrid mode because direct access to clinical sites and patients is reduced.	Monitoring related pediatric-specific methodological training will be needed. For pediatric studies, the items to be closely reviewed can be prespecified.
Trainings	Technology should support adequate training for all stakeholders involved in the DCTs. This includes but is not limited to, participants, site staff, call centers, sponsor staff and provision of insight to regulators and EC/IRBs.	Can be similar to a fully decentralized trial.	There should be specialists available to address issues related to pediatric patients using technologies, such as DHT device placement.

## Electronic supplementary material

Below is the link to the electronic supplementary material.

Supplementary Material

## Data Availability

Data sharing is not applicable to this article as no datasets were generated or analyzed during the current study.

## List of abbreviations

BYOD: Bring Your Own Device
COA: Clinical Outcome Assessment
CRA: Clinical research associate
CRO: Contract Research Organization
DCT: Decentralized Clinical Trials
DHT: Digital Health Technologies
DIA: Drug information Association
DiME: Digital Medicine Society
eCOA: Electronic Clinical Outcome Assessment
eCRFs: Electronic Case Report Forms
EMA: European Medicines Agency
eTMF: electronic Trial Master File
EU: European Union
FDA: Food and Drug Administration
HCP: Healthcare Professional
HIPPA: Health Insurance Portability and Accountability Act
ICE: Intercurrent Event
IDSWG: Innovative Design Scientific Working Group
IMP: Investigational Medical Product
IND: Investigational New Drug
IRB: Institutional Review Board
ISTAND: Innovative Science and Technology Approaches for New Drugs
MRI: Magnetic Resonance Imaging
NEED: Nature and Extent of Evidence required for regulatory Decision
RCT: Randomized Clinical Trial
REMOTE: Research on Electronic Monitoring of Overactive Bladder Treatment Experience
SAP: Statistical Analysis Plan
