# Supplementary material for: Decentralized clinical trials and rare diseases: a Drug Information Association Innovative Design Scientific Working Group (DIA-IDSWG) perspective
Source: Orphanet J Rare Dis. 2023 Apr 11;18:79. doi: 10.1186/s13023-023-02693-7 (PMC10088572; doi:10.1186/s13023-023-02693-7)
Supplement: Supplementary file 1 — Supplementary Material [file 13023_2023_2693_MOESM1_ESM.docx]

# Decentralized Clinical Trials and Rare Diseases: A DIA-Innovative Design Scientific Working Group (DIA-IDSWG) Perspective

Appendix A. List of Released Decentralized Clinical Trials

**Table A1: Examples of published DCTs**

| **Year** | **CT.gov Identifier** | **Disease** | **Study Length** | **Study Design** | **Sample Size** | **Special Comment** | **Insights for Future Studies** |
| --- | --- | --- | --- | --- | --- | --- | --- |
| 2021 | NCT04923464 | Cystic Fibrosis | 12 weeks | Phase IV Decentralized Pilot Study | 51 | Physical activity and cough were measured using wearable devices during at home visits. | - Fit-for-purpose devices should be used. E.g. Actigraphy for activity/sleep, and ambulatory cough monitoring system for cough detection - It is crucial to evaluate the compliance with the wearable devices |
| 2020 | NCT04644315 | Locally-Advanced or Metastatic ALK-Positive Solid Tumors | 5 years | Phase II open-label, single-arm trial | 50 anticipated, with only 1 recruited | Home-based assessments every 4 weeks; Tumor assessments every 8 weeks at local facilities; Oral drug, administered at home, IMP accountability performed by a mobile nurse; Investigator does not see the patient in person (telemedicine). Mobile nurse is at home with the  participant. Local physicians provide continuity of care. Study Terminated due to data no longer needed | A hybrid model with   - Remote: home based assessments, mobile nurse, telemedicine - Tumor assessments at local facilities |
| 2020 | NCT04308668 | COVID 19 (SARS-Coronavirus-2) | 14 days | Phase III, Randomized, double-blind, placebo-controlled trial | 1312 | To limit the spread of disease, no in-person visits with study personnel will be performed. During recruitment and follow-up, most participants will be at home in self-isolation or quarantine | - Screening, informed consent, randomization, and online data collection through the Research Electronic Data Capture (REDCap) - Study medications were delivered to the participants’ residential address using courier service - Questions/concerns were addressed by phone or email with timely responses (same day during business hours and the following day after 9 pm) - Website created to provide live enrollment updates, responses to FAQs, and trial information |
| 2019 | NCT04091087 | Stasis Dermatitis | 14 weeks | Phase II, randomized, double-blind, vehicle-controlled, parallel-group | 66 | Study enrollment and management will be decentralized, where participants do not visit an investigator or a clinic for clinical assessment. The participants will participate in the study at home. The sponsor (or designee) will provide home visits by qualified home visit practitioners (HVP), remote contact by telemedicine (or telephone), and clinical database electronic case report forms (eCRFs), eDiary, and other electronic data entries from 3rd party vendors for study data collection. | A fully decentralized model with:   - Decentralized enrollment and management - Home visits by qualified HVP and telemedicine - 3^rd^ party vendors provided the collection and management of remotely collected eCRF data, eDiary, etc |
| 2018 | NCT03538262 | Parkinson's disease | 2 years | Prospective observational study | 270 | Video visits with a movement disorder specialist, smartphone-based assessments at home, and patient-reported outcomes on an online platform | - Leveraged smartphone-based assessments. - Showed the promise in conducting Movement Disorders Society Unified Parkinson's Disease Rating Scale (MDS-UPDRS) tests (some parts, which are typically collected on site) over telemedicine. |
| 2022 | NCT04862143 | Breast cancer (PIK3CA mutation) | 12 months | Phase II, single-arm, open-label, multicenter | 20 | Primary objective will be to assess participant satisfaction with the decentralized clinical trial (DCT) experience. | This study is a good example of a pilot study to evaluate in a specific disease population:   - Patients’ overall feeling about DCT (satisfaction, retention) - Compliance to drug within a DCT - Whether there will be more than expected un-scheduled clinical visits.   The results from this study can inform the design and conduct of future DCTs in similar patient cohorts. |
| 2016 | NCT02832063 | Mild to moderate Acne Vulgaris | 16 weeks | Phase IIb/III, randomized, double blinded | 372 | Lesion counting was performed using the digital photos taken by participants and stored inside the study mobile platform by trained personnel only | - eConsent obtained at the patient's home. Patients who had no access to a computer/tablet will be shipped a tablet to complete the eConsent - The use of NORA (network-oriented research assistant) app, which is a commonly used tool as a telemedicine platform and digital photography functionality to provide the count of lesions and scoring of disease severity. - The NORA has to be used in provisioned iPhone.   Digital photograph requires consistent input, therefore, using a provisioned phone instead of patients’ own phones will potentially reduce the bias created by the quality of the photos |
| 2012 | NCT01694667 | Autism spectrum disorder | 6 weeks | Double-blinded, randomized placebo-controlled trial | 57 | Internet-based randomized controlled trials of therapies in children with ASD | - Screening, informed consent baseline and outcome assessments were done online. - Participating parents received follow-up emails weekly to report medication adherence, new medical problems, and outcome assessments. - The data collection system met U.S. Food and Drug Administration (FDA), 21 CFR Part11, HL7, and Health Insurance Portability and Accountability Act (HIPAA) compliance criteria for the capture and security of electronic data |
| 2011 | NCT01302938 | Overactive bladder | 12 weeks | Phase IV, randomized, placebo-controlled | 283 | The REMOTE trial is the first entirely web-based trial conducted under an investigational new drug application. | - Participants were recruited via the web, screened for eligibility using web-based questionnaires. - Informed consent was obtained using an interactive web-based method with physician countersignature. - Study medication was shipped directly to participants. - All study data collected, including web-based and mobile phone-based electronic patient-reported outcomes and central laboratory results, were transferred to an electronic data capture-based database.   Some of the good practices:   - A dedicated team to respond to patients’ questions and concerns 24-7 - Prior to signing the informed consent document, each participant was required to pass a multiple-choice test confirming the individual's understanding of the informed consent document. - To minimize potential fraud, approval was obtained from eligible participants to perform a secure and confidential third-party online identity verification using personal information |

Appendix B. Additional Information About Project Planning

**Table B1. Sample questions that can facilitate planning for a Decentralized Clinical Trial (DCT)**

| 1. What level of decentralization is required? | - Full DCT - Hybrid DCT |
| --- | --- |
| 1. What procedures are done at the central facility? | - Blood Draw - Vital sign measurements - Drug distribution |
| 1. What procedures are performed by local or mobile health care? | - Blood Draw - Vital sign measurements - Drug distribution |
| 1. Is mobile technology used for collecting data? | - Yes - No |
| 1. If yes, what data would be collected using mobile technology? | - Patient’s questionnaire - Patient’s heart rate - Patient’s activity intensity |
| 1. What device is proper with this study? | - Actigraph - Fitbit - iPhone |
| 1. How often activity data would be collected? | - 7 days at the baseline and end of treatment each - 5 days at the baseline and end of treatment each |
| 1. What SOPs require modifications? | - SOP1 - SOP2 |
| 1. What training materials need to be modified? | - Patient’s training material - Institution or health care provider’s training material |
| 1. What does study compliance and adherence mean? | - Provided at least 10 hours for 3 days at baseline and the end of treatment |
| 1. What data privacy regulations should be implemented? | - USA-FDA - Canada - Healthcare - EU |
| 1. What tasks are outsourced? | - Data monitoring - Passive Data collection - Site initiation and closing |
| 1. What tasks are internally handled? | - Data monitoring - Passive Data collection - Site initiation and closing |
| 1. Where is data stored and processed? | - Local servers - CRO platform - Cloud |
